# Supplementary material for: Polymerization driven monomer passage through monolayer chemical vapour deposition graphene
Source: Nat Commun. 2018 Oct 3;9:4051. doi: 10.1038/s41467-018-06599-y (PMC6170411; doi:10.1038/s41467-018-06599-y)
Supplement: Supplementary file 1 — Supplementary Information [file 41467_2018_6599_MOESM1_ESM.pdf]

Supplementary Information

Polymerization Driven Monomer Passage through Monolayer CVD Graphene

Zhang et al.

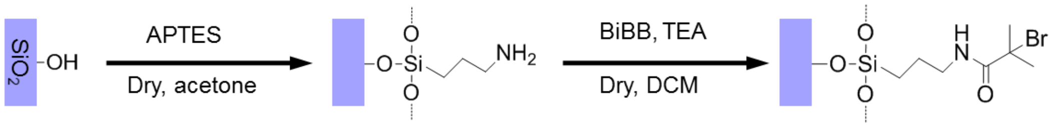

Supplementary Figure 1 | Immobilization of initiator monolayer on SiO_2 wafer. The SiO_2 wafer was firstly functionalized by monolayer 3-aminopropyltriethoxysilane (APTES) in dry acetone, and then in dry dichloromethane (DCM) by monolayer 2-bromoisobutryl bromide (BIBB) as radical initiator for SI-CRP.

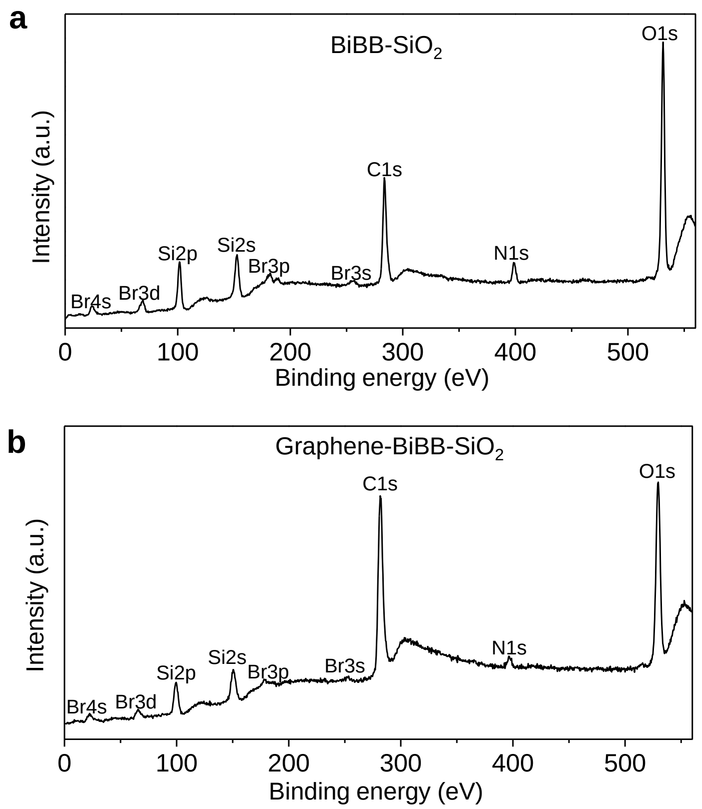

Supplementary Figure 2 | X-ray photoelectron spectroscopy (XPS) survey spectra. (**a)** Monolayer BiBB on SiO_2 substrate. (**b**) Monolayer graphene on BiBB-SiO_2. The N1s peak at the binding energy of about 402.1 eV is attributed to the O C-N species. The presence of the Br3d peak at the binding energy of about 68.7 eV and the appearance of the O C-N peak component on both samples confirmed the immobilization of initiator.

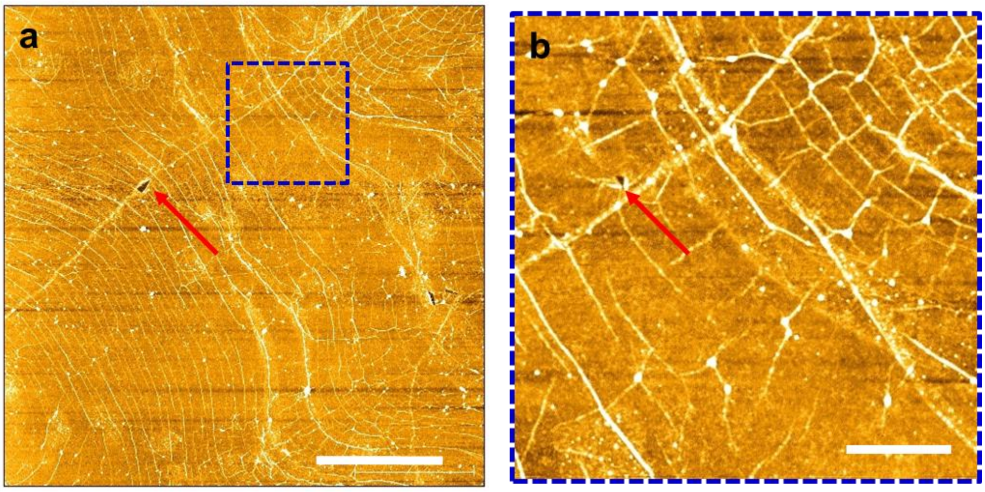

Supplementary Figure 3 | Atomic force microscope (AFM) characterization. (**a**) AFM topographic image of CVD graphene transferred on BiBB-SiO_2 wafer. (**b**) A close-up of (**a**). Scale bars in: (**a**) 5 µm; (**b**) 1 µm.

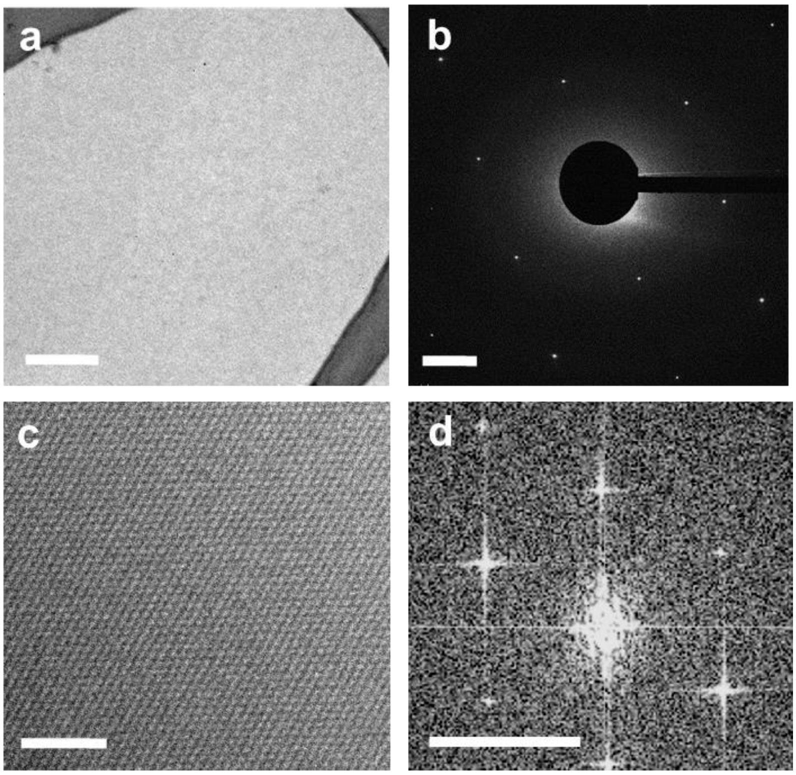

Supplementary Figure 4 | Transmission electron microscope (TEM) characterization. (**a**) Bright-field TEM image of a monolayer graphene membrane suspended over a micro grid. (**b**) The selected area electron diffraction pattern (SAED) taken from the membrane in (**a**). (**c**) HRTEM image of graphene sheet selected from (**a**). (**d**) Corresponding fast Fourier transform (FFT) of (**c**). Scale bars in: (**a**) 200 nm; (**b**) 2 1/nm; (**c**) 2 nm; (**d**) 5 1/nm.

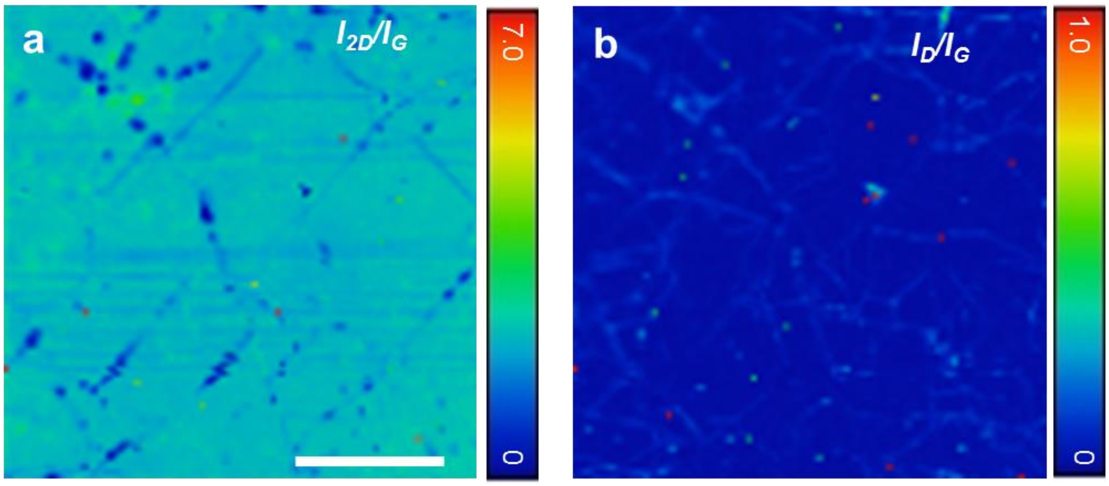

Supplementary Figure 5 | Raman characterization. (**a**) Raman intensity ratio ($I_{2D}/I_G \approx 2.4$) mapping of CVD graphene transferred on BiBB-SiO_2 wafer. Scale bar, 20 μm. (**b**) Corresponding I_D/I_G (< 0.1) mapping of (**a**).

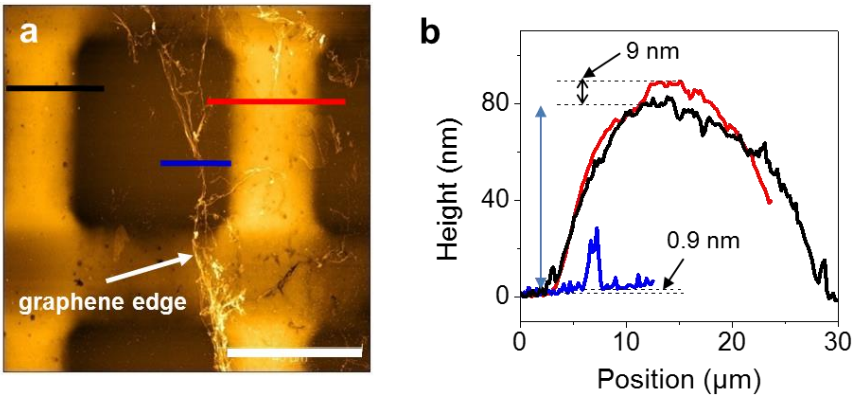

Supplementary Figure 6 | AFM characterization. (**a**) AFM topography image of PMMA brushes at graphene edge, scale bar, 40 μm. (**b**) Height profiles of the black, red and blue lines in (**a**). It implys that monolayer graphene has minor effect on the translocation/polymerization of MMA, since the thickness of PMMA brushes polymerized with and without graphene are identical.

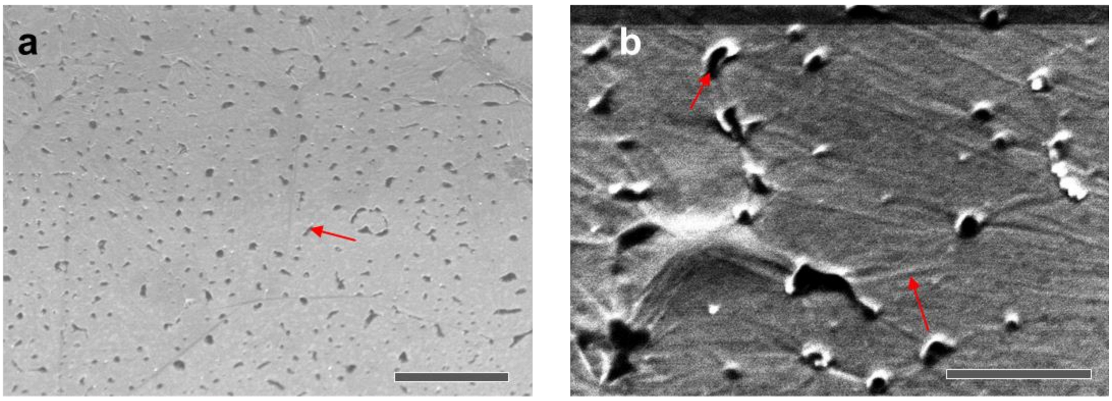

Supplementary Figure 7 | Scanning electron microscope (SEM) characterization. (**a**) SEM of PMMA grafted on graphene-BiBB-SiO_2. (**b**) A close-up of (**a**). These humps correspond to the grain boundary and defects region of graphene. They offered the way for monomer passage though graphene for SI-CRP, resulted in a preference of brush growth at these regions. Scale bars: (**a**) 2 μm; (**b**) 500 nm.

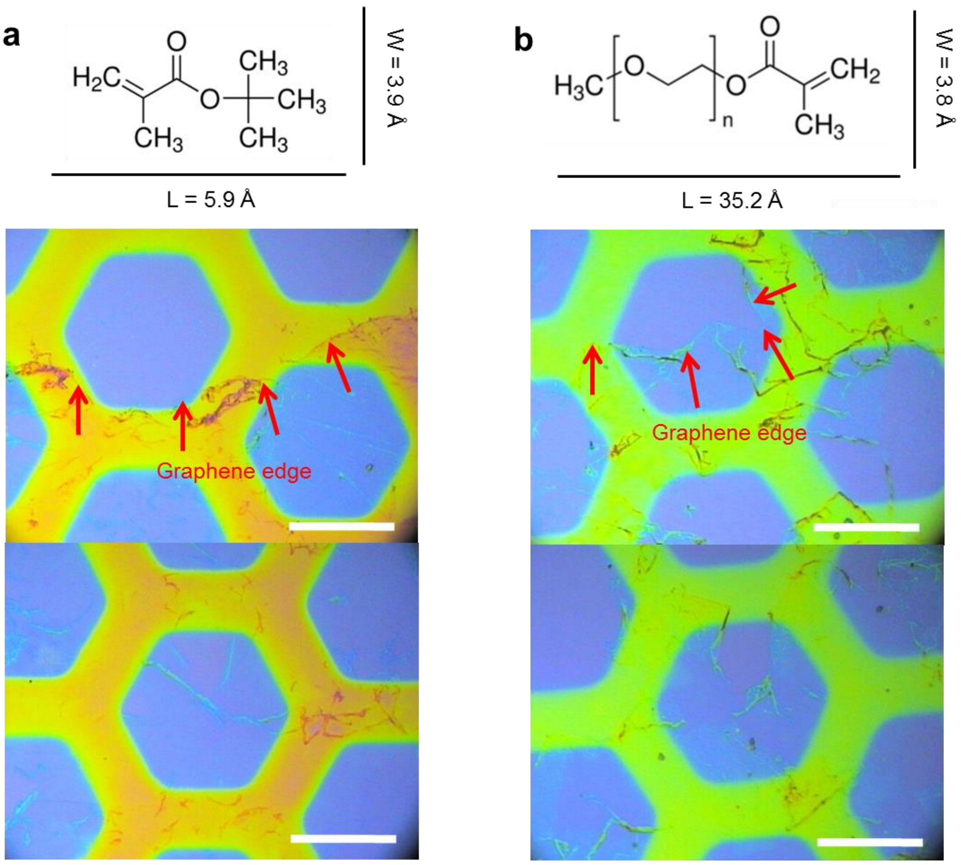

Supplementary Figure 8 | Chemical structures and optical microscopy images. (**a**) The polymer brush under graphene after the translocation of tert-butyl methacrylate (tBuMA). (**b**) The polymer brush under graphene after the translocation of (ethylene glycol) methyl ether methacrylate ($OEGMA_{475}$). Scale bars in (**a**) and (**b**): 40 µm.

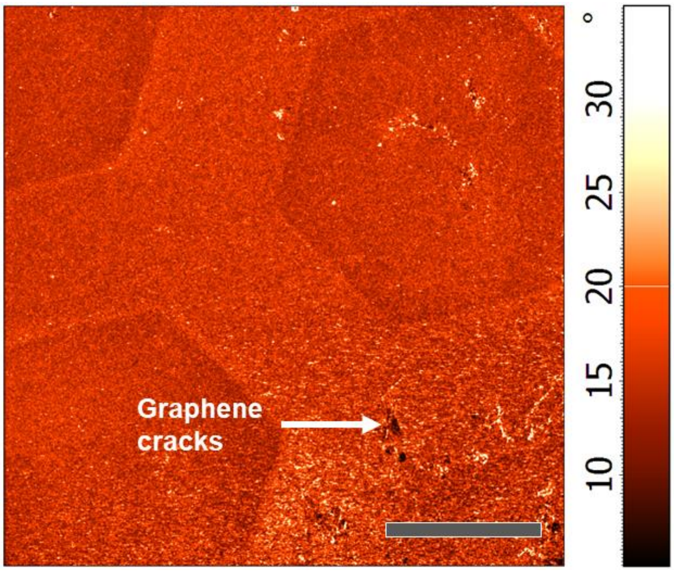

Supplementary Figure 9 | AFM phase image of PMMA brush under graphene. The pattern on the graphene surface is probably due to the increase of defect concertation along the initiator pattern. Scale bar: 20 µm.

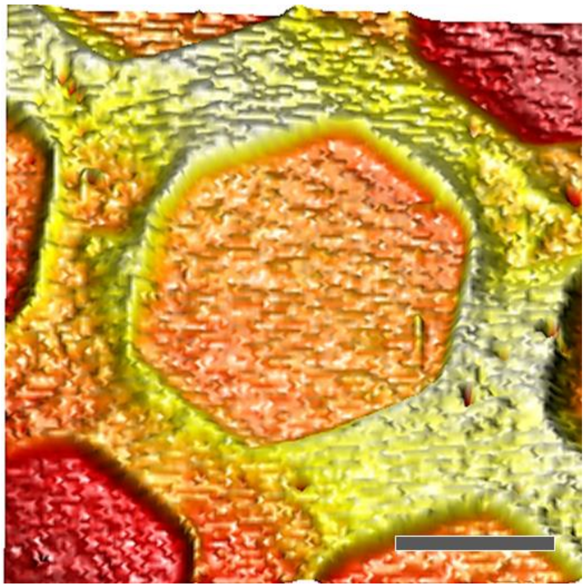

Supplementary Figure 10 | Photomultiplier tube (PMT) image. This image is corresponding to Raman mapping images in Figs. 3d-f. Scale bar: 20 μm.

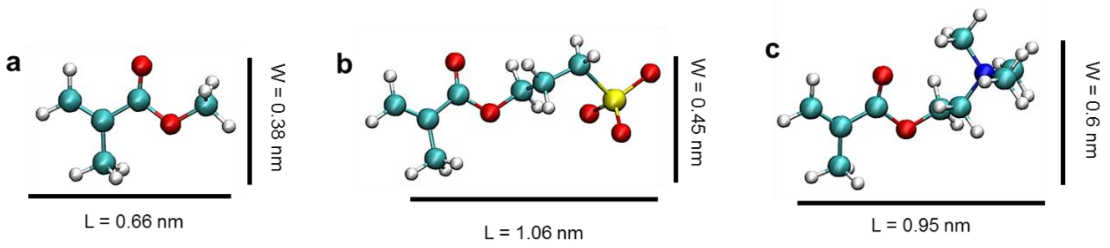

Supplementary Figure 11 | Theatrical size of each monomer. (**a**) MMA. (**b**) SPMA. (**c**) METAC. *L* represents length, and *W* widths.

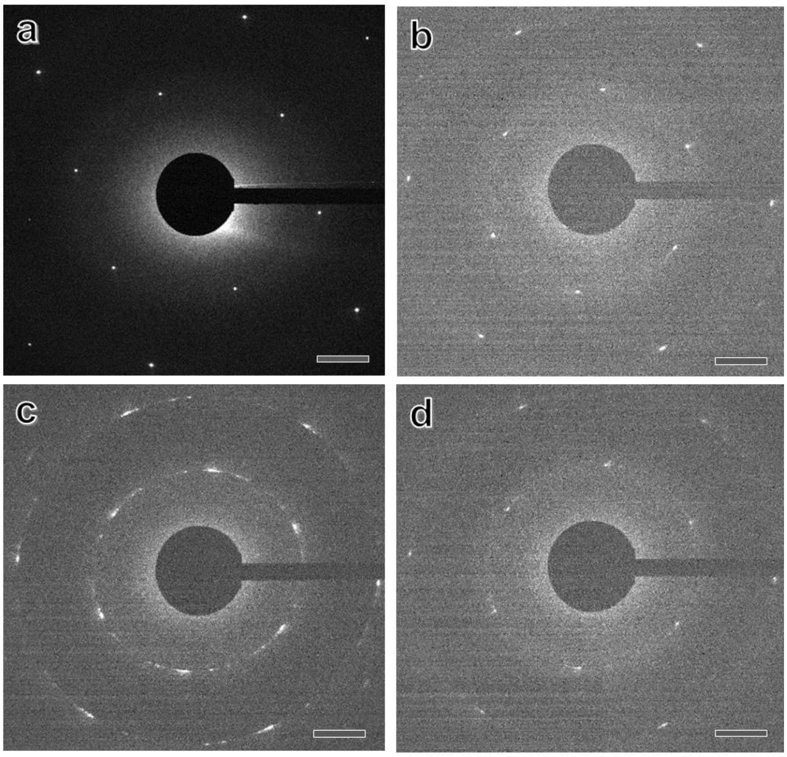

Supplementary Figure 12 | SAED characterization. (**a**) Pristine graphene, and (**b-d**) the graphene after translocation/polymerization of MMA. Please note that: **(b-d**) were taken from different positions of the same sample; a thin layer of PMMA brush is attached below the graphene; the PMMA brush layer is complete amorphous, which is has no effect on SAED, but significant affect high resolution structure imaging. Scale bars: 2 1/nm.

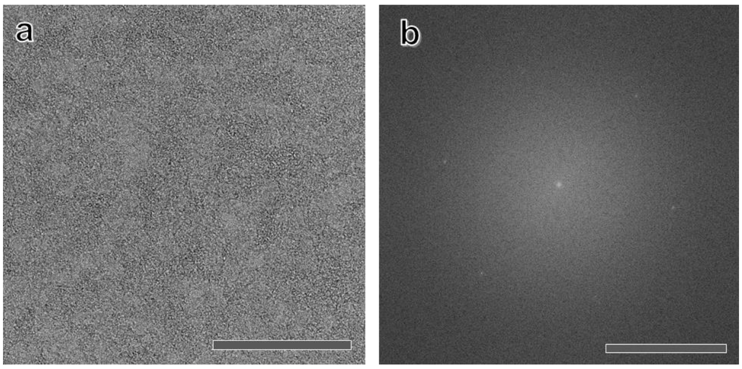

Supplementary Figure 13 | HRTEM and SAED characterization. (**a**) HRTEM image of graphene after MMA translocation. (**b**) Corresponding fast Fourier transform (FFT) of (**a**). Scale bars in: (**a**) 50 nm; (**b**) 5 1/nm. The newly generated nanoscale defects/pores cannot be directly viewed by HRTEM due to the interference of PMMA, and the corresponding fast FFT pattern of characteristic graphene is still visible, but much weaker than pristine graphene.

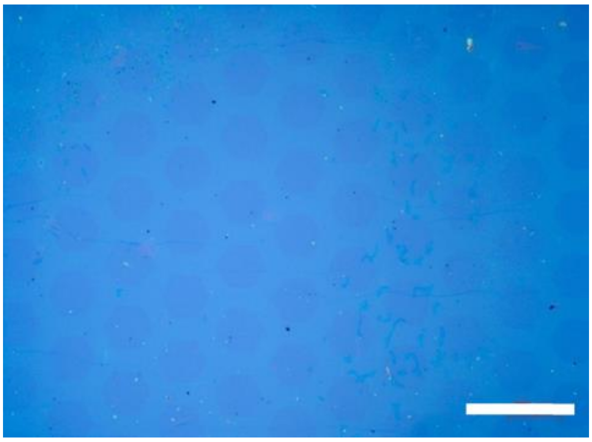

Supplementary Figure 14 | Double-layer graphene after the translocation of MMA. It is clear from the optical microscopy image that there is no PMMA grated on the substrate after polymerization, which is in contrast to the results of monolayer graphene coated BiBB-SiO_2. The hexagon network shows BiBB pattern after UV etching. Scale bar, 80 µm.

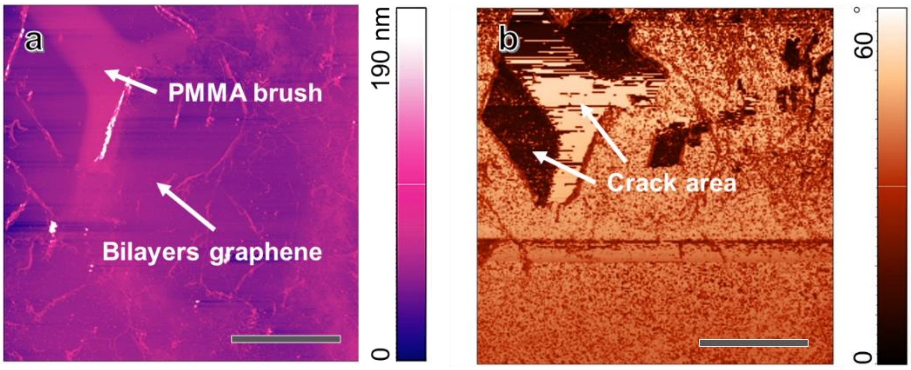

Supplementary Figure 15 | Double-layer graphene after the translocation of MMA. (**a**) AFM topographic and (**b**) phase images of double-layer graphene coated BiBB-SiO_2 after the SI-CRP of MMA. The hexagon network shows BiBB pattern after UV etching. Scale bar, 30 μm. From both topographic and phase images, we can confirm that the PMMA growth on the double-layer graphene covered region was obviously obstructed, which is in clear contrast to the graphene free area (the crack area).

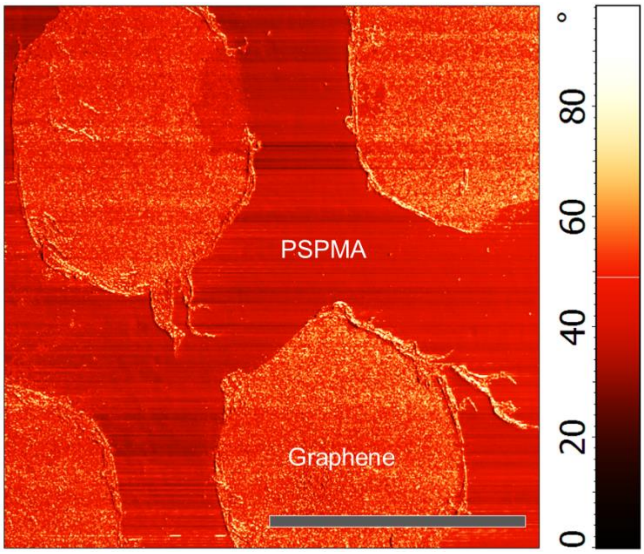

Supplementary Figure 16 | AFM phase image of graphene after translocation of SPMA.

The graphene was “cut” into hexagonal patterns due to the translocation of SPMA driven by SI-CRP. Scale bar: 40 µm.

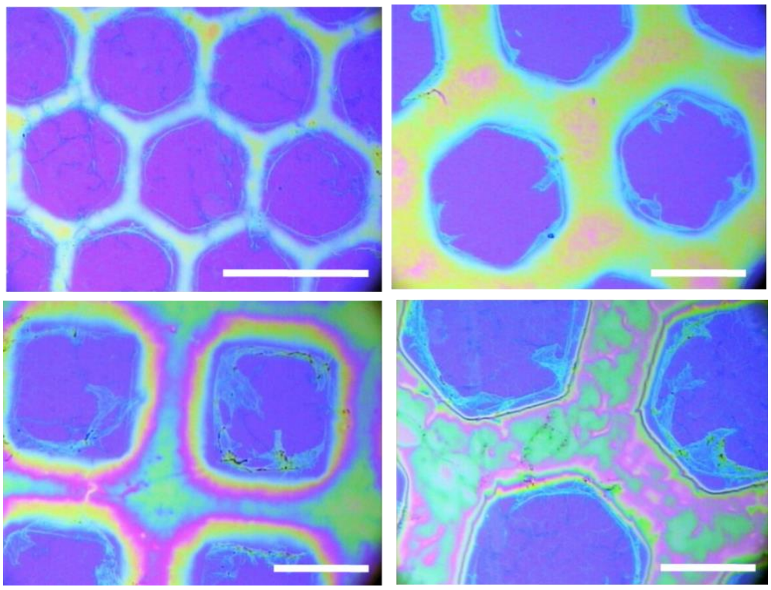

Supplementary Figure 17 | Optical microscopic images of various graphene patterns. Micro-patterned graphene sheet with various size and shape were obtained by the translocation of SPMA through graphene. Scale bars: 40 µm.

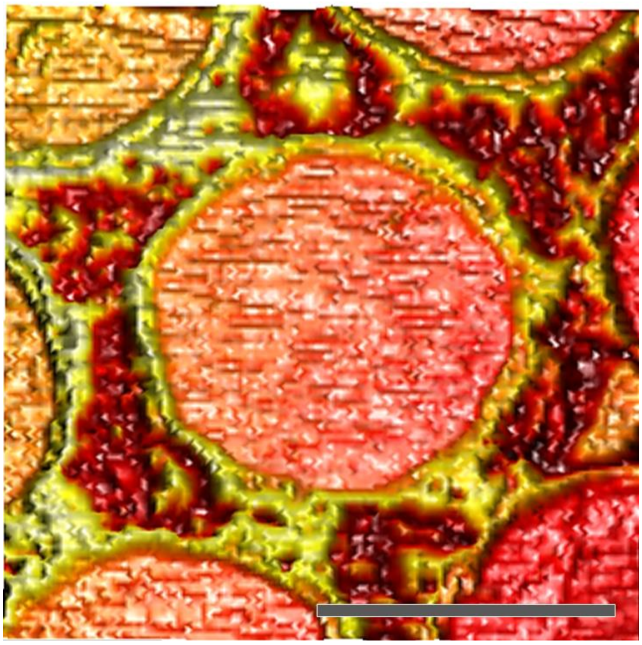

Supplementary Figure 18 | Photomultiplier tube (PMT) image. This image is corresponding to Raman mapping images in Figs. 4d-f. Scale bar: 40 µm.

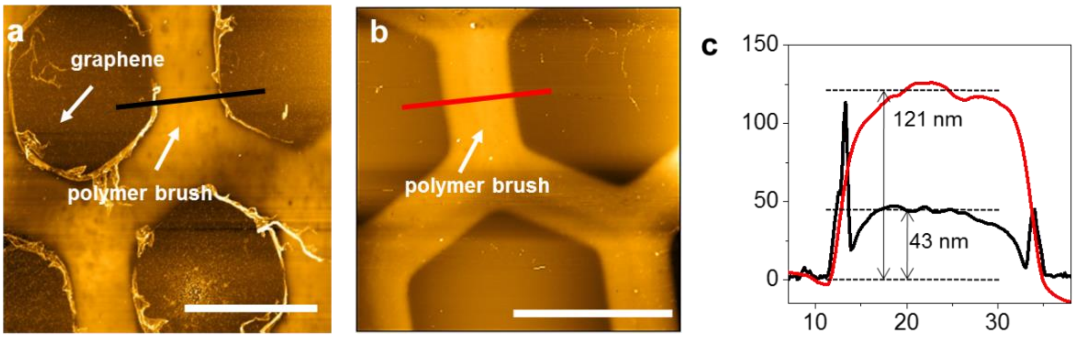

Supplementary Figure 19 | PSPMA and graphene after monomer translocation. (**a**) AFM topography of PSPMA grafted on graphene-BiBB-SiO_2. (**b**) AFM topography of PSPMA grafted on BiBB-SiO_2. (**c**) Height profile of the black and red lines from (**a**) and (**b**), respectively. Scale bars in (**a**) and (**b**): 40 µm.

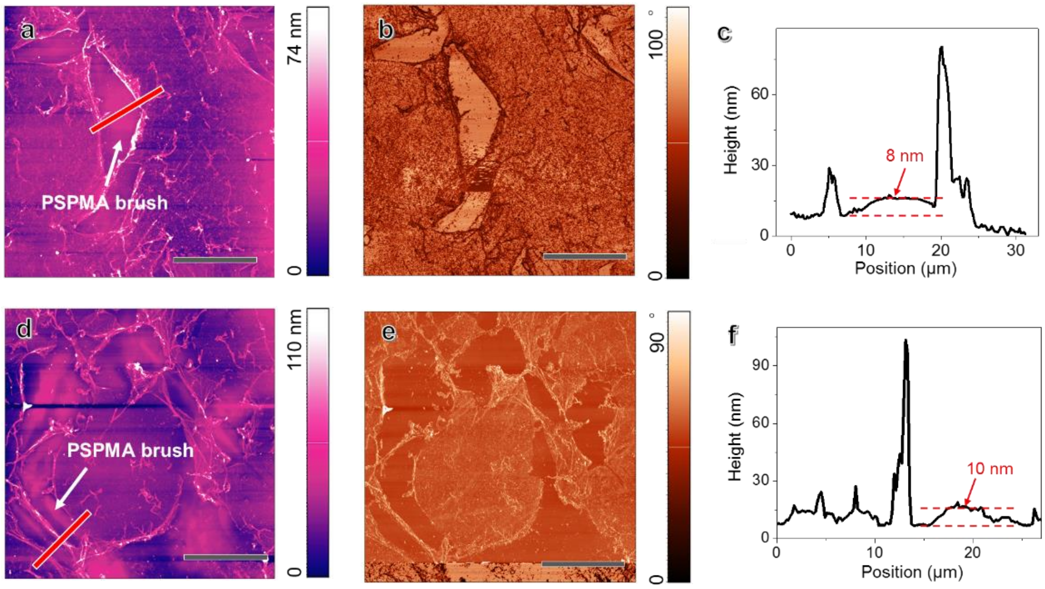

Supplementary Figure 20 | PSPMA and graphene surfaces after 10 mins translocation. Two selected positions of (**a**) AFM topographic and (**b**) phase images, (**c**) corresponding height profile of the red line in (a). (**d**) AFM topographic and (**e**) phase images, (**f**) corresponding height profile of the red line in (d). Scale bars: 30 μm.

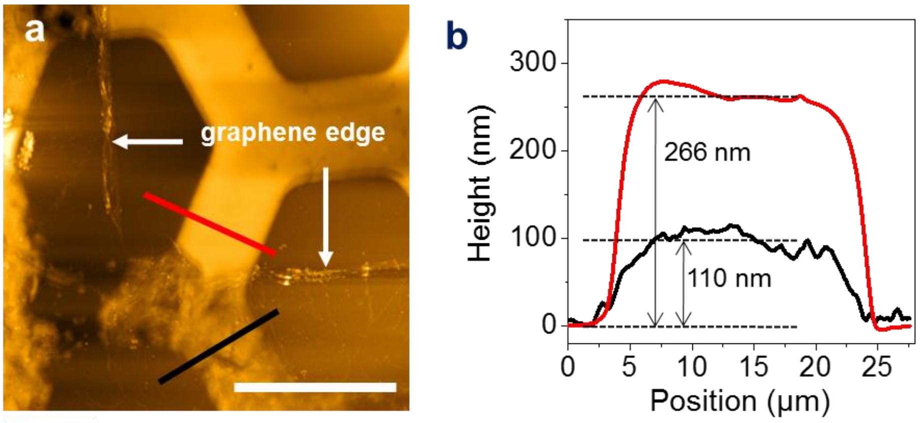

Supplementary Figure 21 | PMETAC under graphene after monomer translocation. (**a**) AFM topography image, scale bar: 40 µm. (**b**) Height profiles of the black and red lines in (**a**). There is a clear contrast on the thickness and morphologies of PMEAC brush with and without graphene coverage.

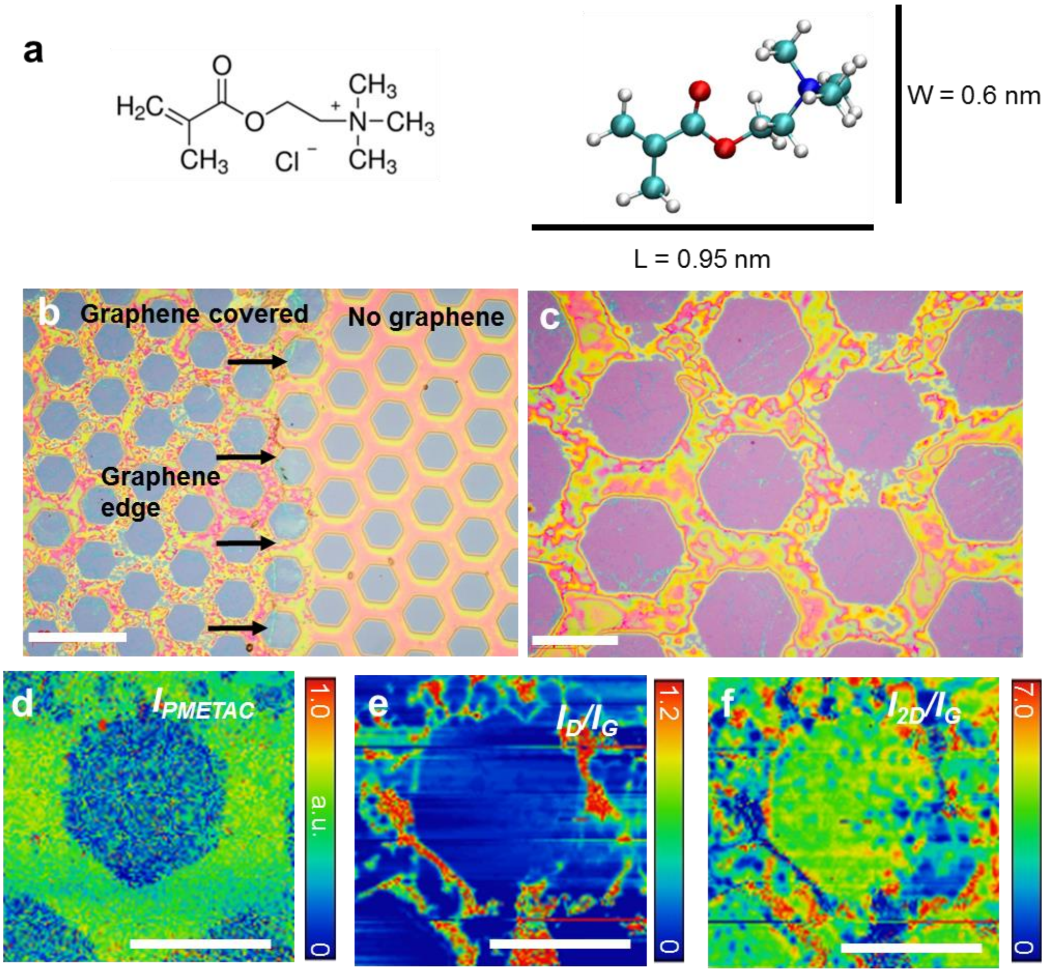

Supplementary Figure 22 | Translocation of METAC through monolayer graphene. (**a**) Molecular structure of METAC. (**b**) Optical image of PMETAC grafted on graphene-BiBB-SiO_2. (**c**) A close-up of (b) of graphene covered region. (**d**) Raman map of integrated intensity of PMETAC. (**e**) Raman mapping integrated intensities ratio of I_D/I_G. (**f**) Raman mapping integrated intensities ratio of I_{2D}/I_G. Scale bars in (**b**): 100 µm; in (**c**-**f**): 40 µm.

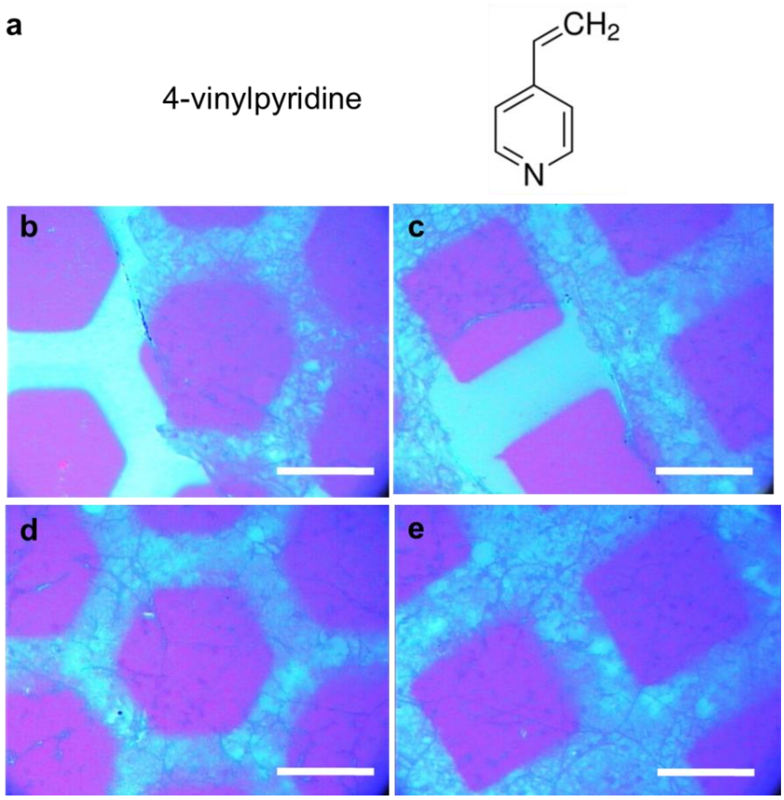

Supplementary Figure 23 | Translocation of 4-vinylpyridine (4VP) through graphene. (**a**) Chemical structure of 4VP. (**b-e**) Optical microscopic images of P4VP grafted on graphene-BiBB-SiO_2 at different positions. Similar to METAC, single-layer graphene has significant interruption on the transport and polymerization of 4VP. Scale bars: 40 µm.

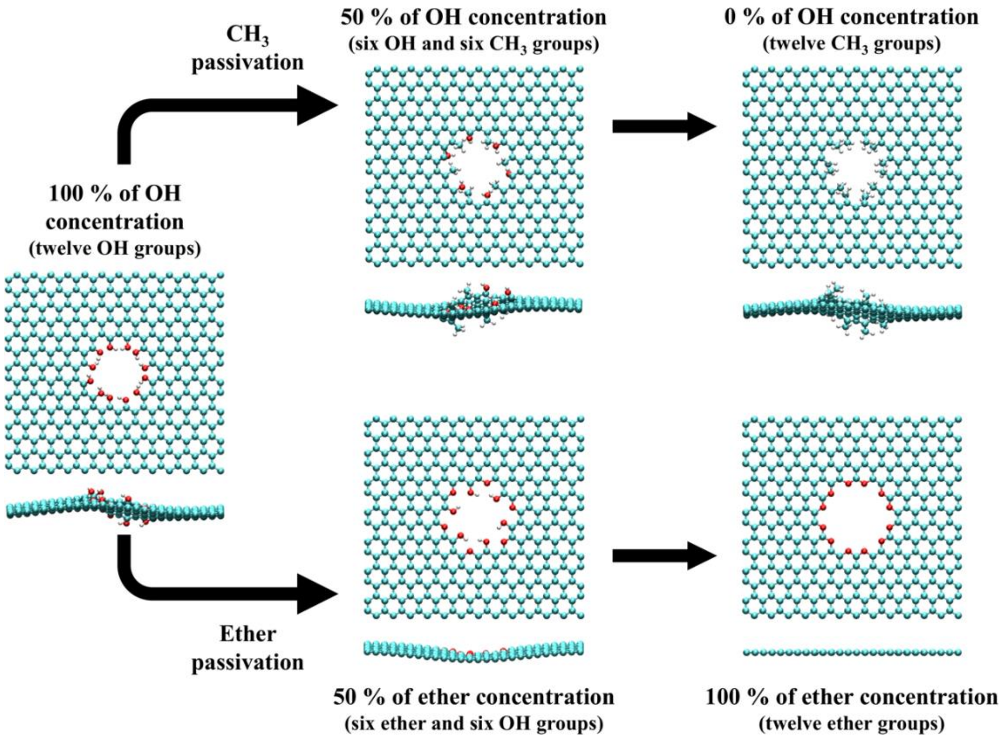

Supplementary Figure 24 | Optimized graphene defects with different terminations. We first generated a graphene pore with CH_3 termination and, hence, the influence of OH termination concentration is studied. Ether termination influence on the translocation process of the monomers through an initially OH-passivated pore has been also analyzed. The dimension of the pore only allows us to consider twelve functional groups for each passivation state.

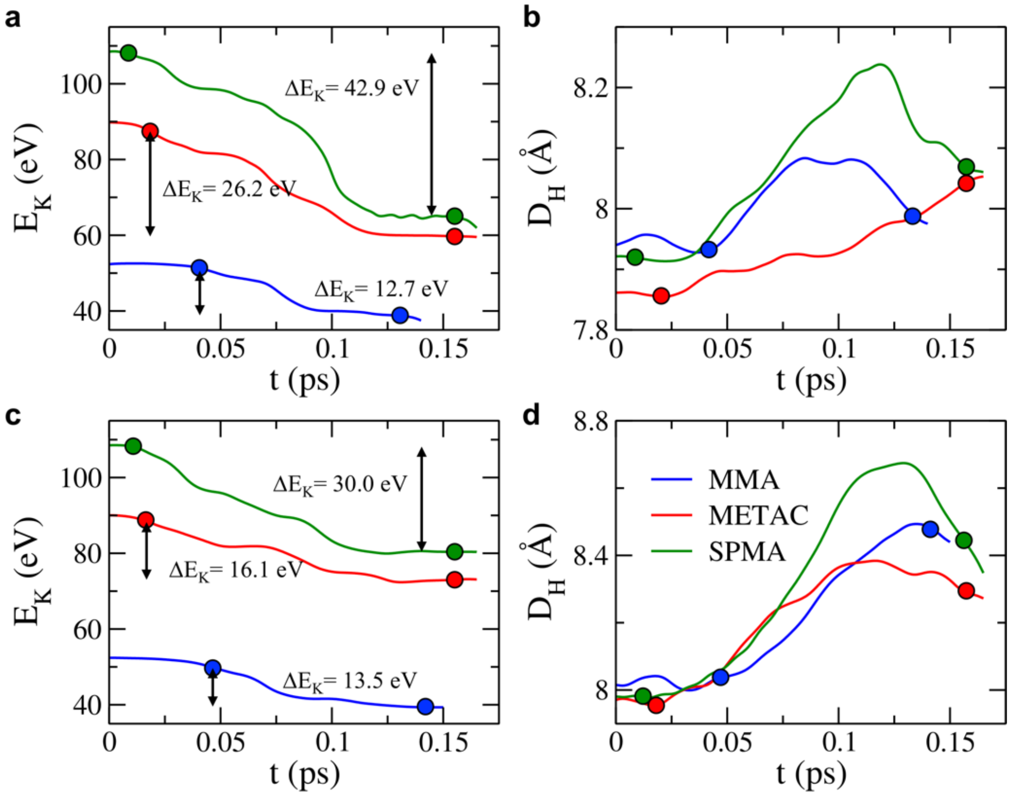

Supplementary Figure 25 | Translocation through OH- and CH_3-passivated pores. Kinetic energy E_K and pore size D_H versus simulation time (**a, b**) fully OH-passivated and (**c, d**) CH_3-passivated pore. The filled circles represent the initial and final time of the translocation process through the pore. The black double-arrows illustrate the change of the kinetic energy ΔE_K of the monomer during the translocation process. As stated in the main text, water-mediated friction effects can be neglected in the discussion. The relevant region to better understand it is the time window between 0 (arbitrarily scaled initial simulation time) and the beginning of the translocation process, i.e. the time window where the monomers do not feel the influence of the nanopore [see panels (a) and (c)]. There, it is clearly seen for the case of the (smaller) neutral monomer (blue solid lines) that the kinetic energy reduction in this region is considerably smaller than after the initiation of the translocation process (e.g. ~1.5 eV vs. ~13.5 eV for the neutral case).

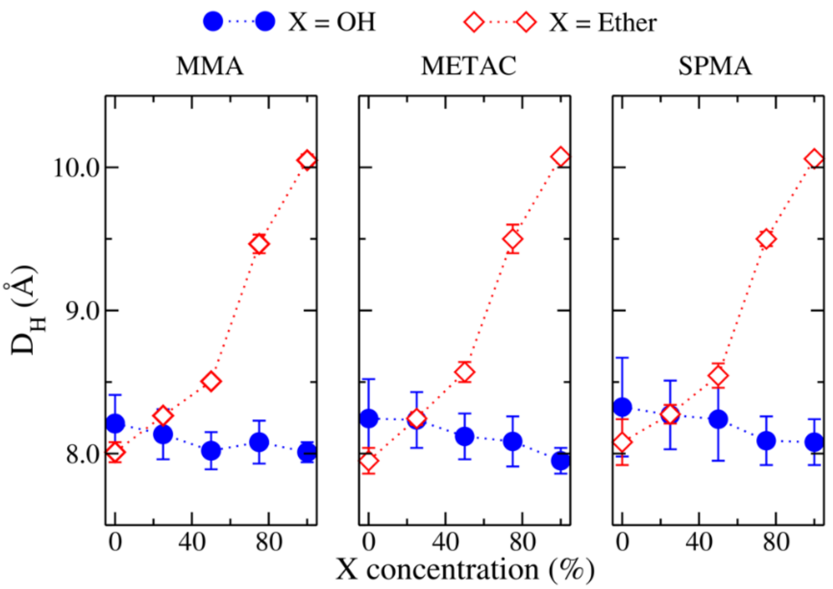

Supplementary Figure 26 | Variation of the pore size D_H as a function of terminations. The monomers MMA (left panel), METAC (central panel), and SPMA (right panel) translocated through the nanopores with various termination *X* (*X* = OH or ether) concentrations from 0 to 100%. The bars at each value represent the range (minimum and maximum pore size value) where the pore size fluctuates during the simulation. To do this, we have considered six different distances among the diametrically opposed functionalization groups in the pore.

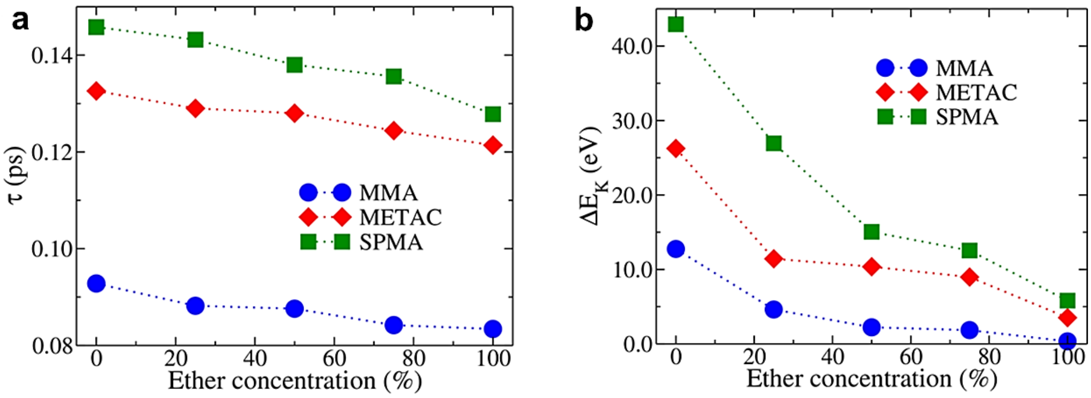

Supplementary Figure 27 | Translocation through ether terminated graphene nanopore. Variation of the (**a**) translocation time τ and (**b**) the change of kinetic energy ΔE_K as a function of the ether concentration for the monomers in the initially OH-passivated defect. $\Delta E_K = E_{K,bef} - E_{K,aft}$, with $E_{K,bef}$ and $E_{K,aft}$ as the kinetic energy of the monomer before and after the translocation process, respectively.

Supplementary Table 1 | Calculated mole of monomer transfer through graphene per m^2 per second.

Monomers	**MMA**	**METAC**	**SPMA**
$M_{m, \text{without graphene}}$ (10^{-7} mol m^{-2} s^{-1})*	1.14	1.64	0.63
$M_{m, \text{with graphene}}$ (10^{-7} mol m^{-2} s^{-1})*	1.01	0.68	0.22
Obstruction effect (%)**	10%	60%	65%

* $M_m = N_m/N_A = P_n/N_A = M_n/M_rN_A = h\rho/\sigma M_r$, $M_n = h\rho N_A/\sigma$, where N_m is the number of monomer transfer per nm^2 per hour; N_A is the Avogadro's number, and ρ is the density of dry polymer (*ca.* 1.2 g per cm^3), and σ = 0.94 chains per nm^2. $M_{r,MMA}$ = 100.12, $M_{r, METAC}$ = 207.70, $M_{r, SPMA}$ = 246.32.

** The value of obstruction effect (%) was obtained from: ($M_{m, \text{without graphene}}$ - $M_{m, \text{with graphene}}$) / $M_{m, \text{without graphene}}$ × 100%.
