# Supplementary material for: Polymerization driven monomer passage through monolayer chemical vapour deposition graphene
Source: Nat Commun. 2018 Oct 3;9:4051. doi: 10.1038/s41467-018-06599-y (PMC6170411; doi:10.1038/s41467-018-06599-y)
Supplement: Supplementary file 3 — Description of Additional Supplementary Files [file 41467_2018_6599_MOESM3_ESM.pdf]

Description of Additional Supplementary Files

File Name: Supplementary Movie 1
Description: Simulation of MMA translocation through a graphene defect.

File Name: Supplementary Movie 2
Description: Simulation of SPMA translocation through a graphene defect.

File Name: Supplementary Movie 3
Description: Simulation of METAC translocation through a graphene defect.
